# Virus-induced interference as a means for accelerating fitness-based selection of cyprinid herpesvirus 3 single-nucleotide variants *in vitro* and *in vivo*

**DOI:** 10.1093/ve/vead003

**Published:** 2023-01-17

**Authors:** Yuan Gao, Arun Sridhar, Noah Bernard, Bo He, Haiyan Zhang, Sébastien Pirotte, Salomé Desmecht, Catherine Vancsok, Maxime Boutier, Nicolás M Suárez, Andrew J Davison, Owen Donohoe, Alain F C Vanderplasschen

**Affiliations:** Immunology-Vaccinology, Department of Infectious and Parasitic Diseases, Fundamental and Applied Research for Animals & Health (FARAH), Faculty of Veterinary Medicine, University of Liège, Liège B-4000, Belgium; Immunology-Vaccinology, Department of Infectious and Parasitic Diseases, Fundamental and Applied Research for Animals & Health (FARAH), Faculty of Veterinary Medicine, University of Liège, Liège B-4000, Belgium; Immunology-Vaccinology, Department of Infectious and Parasitic Diseases, Fundamental and Applied Research for Animals & Health (FARAH), Faculty of Veterinary Medicine, University of Liège, Liège B-4000, Belgium; Immunology-Vaccinology, Department of Infectious and Parasitic Diseases, Fundamental and Applied Research for Animals & Health (FARAH), Faculty of Veterinary Medicine, University of Liège, Liège B-4000, Belgium; Immunology-Vaccinology, Department of Infectious and Parasitic Diseases, Fundamental and Applied Research for Animals & Health (FARAH), Faculty of Veterinary Medicine, University of Liège, Liège B-4000, Belgium; Immunology-Vaccinology, Department of Infectious and Parasitic Diseases, Fundamental and Applied Research for Animals & Health (FARAH), Faculty of Veterinary Medicine, University of Liège, Liège B-4000, Belgium; Laboratory of Animal Genomics, GIGA-Medical Genomics, GIGA-Institute, University of Liège, Liège B-4000, Belgium; Immunology-Vaccinology, Department of Infectious and Parasitic Diseases, Fundamental and Applied Research for Animals & Health (FARAH), Faculty of Veterinary Medicine, University of Liège, Liège B-4000, Belgium; Immunology-Vaccinology, Department of Infectious and Parasitic Diseases, Fundamental and Applied Research for Animals & Health (FARAH), Faculty of Veterinary Medicine, University of Liège, Liège B-4000, Belgium; MRC-University of Glasgow Centre for Virus Research, Glasgow G61 1QH, UK; MRC-University of Glasgow Centre for Virus Research, Glasgow G61 1QH, UK; Immunology-Vaccinology, Department of Infectious and Parasitic Diseases, Fundamental and Applied Research for Animals & Health (FARAH), Faculty of Veterinary Medicine, University of Liège, Liège B-4000, Belgium; Bioscience Research Institute, Technological University of the Shannon, Midlands Midwest, Athlone, Co. Westmeath N37HD68, Ireland; Immunology-Vaccinology, Department of Infectious and Parasitic Diseases, Fundamental and Applied Research for Animals & Health (FARAH), Faculty of Veterinary Medicine, University of Liège, Liège B-4000, Belgium

**Keywords:** herpesvirus, alloherpesvirus, virus evolution, fitness-based selection, purifying selection

## Abstract

Cyprinid herpesvirus 3 (CyHV-3) is the archetype of fish alloherpesviruses and is advantageous to research because, unlike many herpesviruses, it can be studied in the laboratory by infection of the natural host (common and koi carp). Previous studies have reported a negative correlation among CyHV-3 strains between viral growth *in vitro* (in cell culture) and virulence *in vivo* (in fish). This suggests the existence of genovariants conferring enhanced fitness *in vitro* but reduced fitness *in vivo* and *vice versa*. Here, we identified the syncytial plaque formation *in vitro* as a common trait of CyHV-3 strains adapted to cell culture. A comparison of the sequences of virion transmembrane protein genes in CyHV-3 strains, and the use of various recombinant viruses, demonstrated that this trait is linked to a single-nucleotide polymorphism (SNP) in the open reading frame (ORF) *131* coding sequence (*C225791T* mutation) that results in codon 183 encoding either an alanine (183A) or a threonine (183T) residue. In experiments involving infections with recombinant viruses differing only by this SNP, the 183A genovariant associated with syncytial plaque formation was the more fit *in vitro* but the less fit *in vivo*. In experiments involving coinfection with both viruses, the more fit genovariant contributed to the purifying selection of the less fit genovariant by outcompeting it. In addition, this process appeared to be accelerated by viral stimulation of interference at a cellular level and stimulation of resistance to superinfection at a host level. Collectively, this study illustrates how the fundamental biological properties of some viruses and their hosts may have a profound impact on the degree of diversity that arises within viral populations.

## Graphical Abstract

**Figure:**
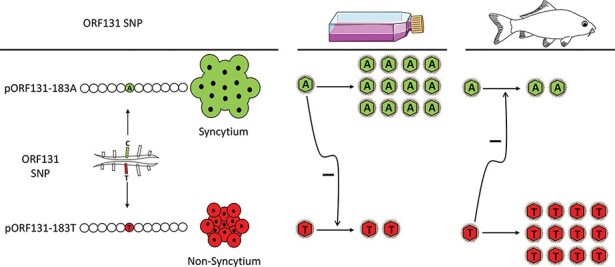

## 1. Introduction

The order *Herpesvirales* comprises large, double-stranded DNA viruses classified in three distantly related families: *Herpesviridae* (herpesviruses of reptiles, birds, and mammals), *Malacoherpesviridae* (herpesviruses of mollusks), and *Alloherpesvirida*e (herpesviruses of amphibians and fish) (Hanson, Dishon, and Kotler 2011). Most members of the family *Alloherpesviridae* (referred to as alloherpesviruses) have been recognized because they cause disease outbreaks associated with mass mortalities that have a serious economic impact on the aquaculture sector.

Current knowledge of herpesvirus evolution relies almost entirely on the study of members of the family *Herpesviridae*. These studies suggest that, despite the proof-reading activity of herpesvirus DNA polymerases, the large size of herpesvirus genomes facilitates a degree of tolerance toward genetic drift (e.g. accumulation of single-nucleotide polymorphisms (SNPs) and insertions/deletions (indels)) in successive rounds of viral replication (Renner and Szpara 2017). Larger-scale changes due to genetic shift also occur, with inter-strain recombination resulting in individual strains acquiring combinations of advantageous genes or genovariants initially present in different parental genomes (Umene 1999; Kolb et al. 2017; Law et al. 2018). This process requires coinfection of the same host cell by two parental viruses and can result either from simultaneous infection or from infection by one strain followed by superinfection by another. The diversity generated by genetic drift and shift is subject to constant fitness-based selection dictated by environmental pressures, which may differ markedly *in vitro* (in cell culture) and *in vivo* (in the natural host). Understanding the key factors that determine how purifying (negative) selection operates on herpesvirus genomes would provide useful insights into the evolution of these viruses.

Compared to the family *Herpesviridae*, there are relatively few studies regarding evolution within the family *Alloherpesviridae* (Waltzek et al. 2005, 2009; Aoki et al. 2007; Davison et al. 2013). Recently, we investigated the diversity of core gene sequences within species clades of the genus *Cyprinivirus* (referred to as cypriniviruses and comprising alloherpesviruses infecting cyprinids and eels) (Donohoe et al. 2021). This revealed significantly less genetic diversity within *Cyprinivirus* species clades than within species in the family *Herpesviridae*, which may be linked, at least in part, to different environmental pressures. Cyprinid herpesvirus 3 (CyHV-3) is the archetypal *Cyprinivirus* (Boutier et al. 2015b). This virus can be studied in the laboratory by infection of the natural host, the common and koi carp (*Cyprinus carpio* species). Since its emergence in the 1990s, CyHV-3 has caused severe economic loss within the carp culture industry worldwide (Perelberg et al. 2003; Bondad-Reantaso et al. 2005). In addition, it has had an ecological impact by inducing carp mortalities in natural habitats (Rakus et al. 2013; Adamek et al. 2014). Genomic and biological comparisons of CyHV-3 strains have revealed a negative correlation among strains between viral growth *in vitro* and virulence *in vivo* (Gao et al. 2018), suggesting the existence of genovariants conferring advantages *in vitro* but reduced fitness *in vivo* and *vice versa*. The identification of such genovariants would provide a valuable opportunity to study the competition between variants and also the process of directional selection under controlled conditions. In the present study, we identified such genovariants of the CyHV-3 open reading frame (ORF) *131* gene. This gene is essential for viral growth in cell culture and encodes a 429-amino acid (aa) type 1 membrane protein with the vast majority of the protein (aa23–391) predicted to be expressed on virion envelope (Vancsok et al. 2017). The CyHV-3 *ORF131* gene is encoded on the reverse orientation of the CyHV-3 genome and consists of three exons. The third exon encodes an aa sequence that is closely related to an uncharacterized type 1 membrane protein found in various cyprinid fish species. Orthologs of CyHV-3 *ORF131* are found in Cyprinid herpesvirus 1 (CyHV-1) and Cyprinid herpesvirus 2 (CyHV-2). They are also predicted to encode type 1 membrane proteins, but they lack the region that corresponds to the first two exons of CyHV-3 *ORF131* and consequently are not predicted to be spliced. All three cyprinid herpesvirus *ORF131* orthologs encode two threonine-rich regions indicative of O-linked glycosylation. The function of the ORF131 protein is unknown. No *ORF131* homologs were reported in other alloherpesviruses or in the family *Herpesviridae*.

In the present study, we first identified syncytial plaque formation *in vitro* as a common trait of CyHV-3 strains exhibiting greater replicative fitness in cell culture. Next, we demonstrated by mutagenesis that the genetic determinant of this trait depends on an SNP in the *ORF131* coding sequence (*C225791 T* mutation) that results in codon 183 encoding either an alanine (183A) or a threonine (183 T) residue. Strains adapted to cell culture encode an alanine residue at codon 183 (the 183A genovariant), whereas field strains encode a threonine residue at this position (the 183T genovariant). Pairs of viruses differing only by the *C225791T* SNP were generated and compared for fitness *in vitro* and *in vivo* by infection with single viruses or coinfection with both viruses. These experiments demonstrated the higher fitness of the 183A genovariant *in vitro* and the 183T genovariant *in vivo*. Interestingly, we found that purifying selection of the least fit variant occurs rapidly during lytic coinfections both *in vitro* and *in vivo*. When more than one virus attempts to enter or initiate replication in the same cell or host organism, a phenomenon referred to as interference can occur, where one virus negatively impacts the replication (and by extension, fitness) of the other. This is a very general, widespread, and well-known phenomenon among viruses (Henle 1950; Remion et al. 2013; Schultz-Cherry 2015; Kumar et al. 2018a; Escobedo-Bonilla 2021; Piret and Boivin 2022). In this study, further investigations revealed that purifying selection of the least fit variant could, at least in part, be facilitated by the onset of interference at a cellular level *in vitro* and increased resistance to superinfection at a host level *in vivo*. This study illustrates how the host–virus interactions and the fundamental biological properties of some viruses and their hosts may have a profound impact on the degree of diversity that arises within viral populations.

## 2. Materials and methods

### 2.1 Cells and viruses

Common carp brain (CCB) cells (Neukirch, Böttcher, and Bunnajirakul 1999) were cultured as described previously (Rakus et al. 2017). The seven CyHV-3 strains used had various geographic origins and have been described previously (see Table 1 in Gao et al. 2018).

### 2.2 Indirect immunofluorescence staining

Cells grown on glass coverslips were fixed in phosphate buffered saline (PBS) containing 4 per cent (wt/v) paraformaldehyde (PAF) at 4°C for 15 min and then 20°C for 10 min. After washing with PBS, samples were permeabilized in PBS containing 0.1 per cent (v/v) Nonidet P40 at 37°C for 15 min. Immunofluorescence staining (incubation and washes) was performed in PBS containing 10 per cent (v/v) fetal calf serum (FCS). The monoclonal antibody 4B5 against the CyHV-3 protein encoded by *ORF65* (pORF65) was used as a primary antibody (dilution 1:1,000). Alexa 488 goat-anti-mouse immunoglobulin G (H+L) (Invitrogen) was used as a secondary antibody (dilution 1:1,000). After washing, the cells were mounted using Prolong Gold antifade reagent with 4ʹ,6ʹ-diamidino-2-phenylindole (DAPI; Invitrogen).

### 2.3 Confocal microscopy and image analysis

Specimens were analyzed by confocal microscopy using a Leica SP5 or Nikon A1R instrument, and the results were compiled using ImageJ software (Abramoff, Magalhães, and Ram 2004).

### 2.4 DNA sequence alignment

Multiple DNA sequence alignments were made using MAFFT online version 7 (Katoh, Rozewicki, and Yamada 2019) and then processed using MEGA X software (Kumar et al. 2018b) (https://www.megasoftware.net/).

### 2.5 Production of CyHV-3 FL BAC plasmids and viruses

CyHV-3 strain FL was isolated in Belgium from a fish that died from CyHV-3 infection and used to produce the FL BAC plasmid (Costes et al. 2008), in which the BAC vector is inserted at the 3ʹ end of *ORF55*, which encodes thymidine kinase (TK). The BAC vector includes an enhanced green fluorescent protein (EGFP) expression cassette. Consequently, viruses reconstituted from FL BAC plasmids without the provision of a means to revert the *ORF55* locus to wild type (WT) express a truncated TK from the viral sequence and EGFP from the BAC vector (which is retained in the viral genome). TK truncation was previously shown to have no effect on CyHV-3 growth *in vitro* but reduces virulence slightly *in vivo* (Costes et al. 2008). The FL BAC plasmid also encodes a truncated ORF27 and the ORF131 183A genovariant. FL BAC plasmid derivatives were produced using the two-step positive/negative selection of the *galactokinase* gene (*galK*) in bacteria, and recombinant viruses were generated by co-transfection of these plasmids and, where necessary, recombination cassettes into CCB cells (Boutier et al. 2015a). The strategy is outlined in Figs 2A and 6A, and the primers used are listed in Table 1.

**Table 1.** Oligonucleotide primers.

	Primer name	Sequence (5ʹ–3ʹ)	Coordinates^a^/GenBank Accession no.
Synthesis of recombination cassettes			
Cassette name			
ORF 27 Del *galK*	ORF27 *galK* F	CAGTGTCAAAGAATCATTTTTCTTGACCTGGTAGACTTTTTG TCTGTGTGTACCCCGGTG AATCGTGGTCATATC *CCTGTTGACAATTAATCATCGGCA*	48459–48533
	ORF27 *galK* R	GGTCCTCACCCGAGTACAAGACCAAGCTGTACC CGGGATGGAAGGCGCCGCACAGCTGGGCCTCG GCCGTCGAGA *TCAGCACTGTCCTGCTCCTT*	50155–50229
ORF 131 Del *galK*	ORF131 *galK* F	GTGAGGGAGTGATATGGAGTGAACGTAAAT GGAGGGGCGCTGCGGAGGTT *CCTGTTGACAATTAATCATCGGCA*	225410–225484
	ORF131 *galK* R	TCGAGACGCCCGAACTGGTCGAGGCCTACGTG AACGACGTCAAGGTCCGCTCAGCACTGTCCTGCTCCTT	226426–226352
mCherry	mCherry F	CTTGTACAGCTCGTCCATGC	
	mCherry R	ATGGTGAGCAAGGGCGAGGA	
ORF 131-183T	ORF131-ex-F	CAGCCTGAAGACGATGTCC	225010–225028
	ORF131-ex-R	AGAGTTTCATGAGACGCCAAA	226851–226871
qPCR analysis			
Gene amplified			
CyHV-3 *ORF89*	KHV-86F	GACGCCGGAGACCTTGTG	AF41180.3
	KHV-163R	CGGGTTCTTATTTTTGTCCTTGTT	
	KHV-109P	(6FAM) CTTCCTCTGCTCGGCGAGCACG (BHQ1)	
Carp *glucokinase*	CgGluc-162F	ACTGCGAGTGGAGACACATGAT	AF05333.2
	CgGluc-230R	TCAGGTGTGGAGCGGACAT	
	CgGluc-185P	(6FAM) AAGCCAGTGTCAAAATGCTGCCCACT (BHQ1)	
Genovariant genotyping			
CyHV-3 *ORF131*	ORF131-5F	GTCGGGAGTGGAGTGGTG	225986–226004
	ORF131-5R	CGCAATTTACTGCCATGTGT	226,451-226,470
CyHV-3 *ORF131* SNP	ORF131-A-Rev	/rhAmp-F/AGGAAGAGTTCCTGATACAGGrCCGCC/GT2/^b^	226229–226248
	ORF131-T-Rev	/rhAmp-Y/AGGAAGAGTTCCTGATACAGArCCGCC/GT2/^b^	226229–226248
	ORF131-Fw	GCCGACGTTGATGGTGATGATrGGGTG/GT2/^b^	226141–226159

a Coordinates based on the reference CyHV-3 genome (GenBank Accession no. DQ657948.1). Underlined: sequence not corresponding to the CyHV-3 genome.

Italic: sequence corresponding to *galK.*

b Genovariant-specific primers: The extended 5ʹ sequence of ORF131-A-Rev primer (/rhAmp-F/) is complementary to a universal forward primer and FAM-labeled universal probe for the detection of the ORF131 183A genovariant. The extended 5ʹ sequence of the ORF131-T-Rev primer (/rhAmp-Y/) is complementary to a universal forward primer and a Yakima Yellow-labeled universal probe (detected in HEX channel) for the detection of the ORF131 183T genovariant. GT2 acts a blocking moiety, r denotes an RNA base.

**Figure 1.**
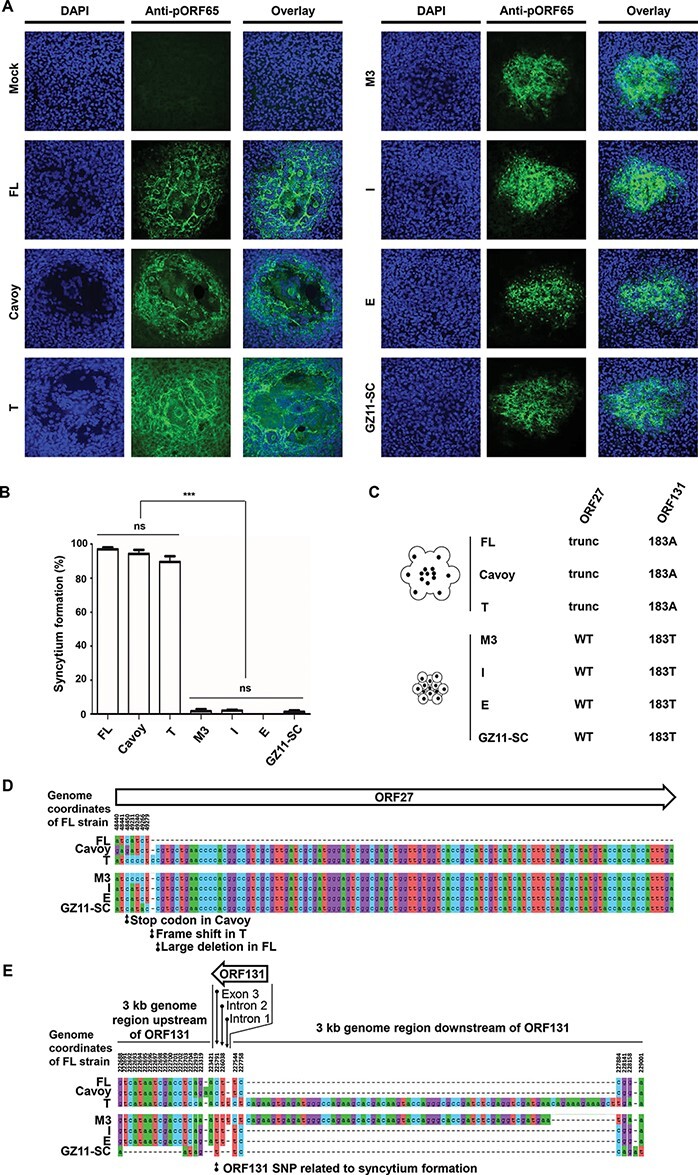
Viral plaque phenotypes and potential genetic determinisms. (A) Viral plaque phenotypes. CCB cells were mock-infected or infected with the CyHV-3 strains indicated and overlaid with CMC in order to obtain isolated plaques. At 3 days PI, the cell monolayers were stained with DAPI and immunostained with an anti-pORF65 monoclonal antibody. Representative plaques are shown. Each panel corresponds to an area of 387.5 µm^2^ from each specimen. (B) Quantification of syncytial plaque formation. One-way ANOVA indicated that the strain (***, *P*< 0.001) had a significant impact on phenotypic observations with pairwise comparison using the Dunnett’s test, indicating that differences were entirely due to lower syncytium formation with M3, I, E, and GZ11-SC strains. The percentage of syncytial plaques was quantified at 3 days PI. The data represent the mean + standard error of the mean (SEM) of triplicate measurements of 100 plaques. (C) *ORF27* and *ORF131* genotypes of the strains tested. trunc, truncation. (D and E) Nucleotide changes observed among CyHV-3 strains in *ORF27* (D) and *ORF131* (E) regions. Multiple sequence alignment encoded by CyHV-3 strains was performed using the FL strain as a reference (MG925487.1). Only nucleotide changes are presented. (E) The SNP correlated with syncytial plaque formation is indicated (*C225791T*), with C occurring at this locus in syncytia-forming strains. As this gene is encoded in the reverse orientation of the CyHV-3 genome, the SNP resulting in a C to T transition in the *ORF131* coding sequence, equivalent to position 225791 in CyHV-3 FL strain (GenBank Accession MG925487.1), can alternatively be categorized as an A to G transition in the forward orientation of the genome. *ORF131*is 1,290 nucleotides and encodes a protein of 429 amino acid residues. The SNP results in codon 183 encoding either an alanine (183A) or a threonine (183T) residue.

**Figure 2.**
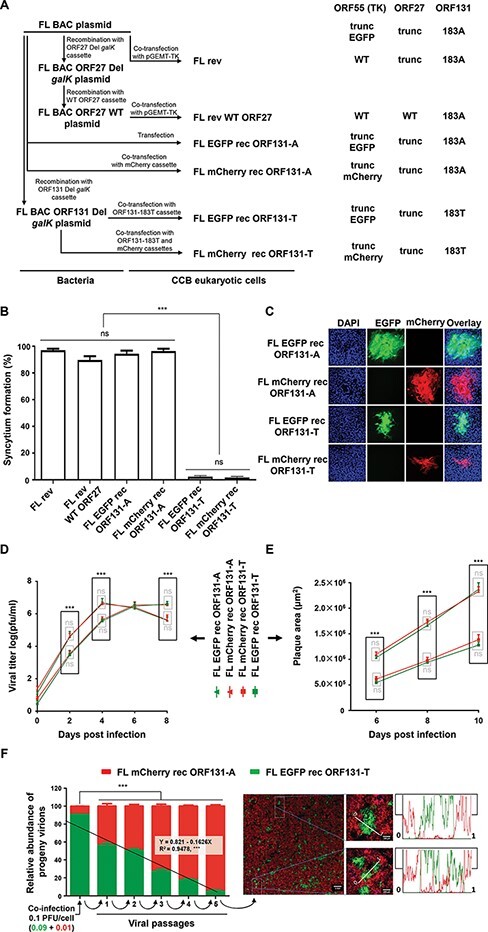
Genetic determinism of syncytial plaque formation. (A) Flow chart of the production of CyHV-3 recombinant viruses. Each virus was assigned the name indicated in the middle part of the panel. The right part of the panel summarizes the genotypes of *ORF55*, *ORF27*, and *ORF131*. Del, deleted; trunc, truncated. (B) Quantification of syncytial plaque formation. The percentage of syncytial plaques was quantified at 3 days PI. The data represent the mean + SEM of triplicate measures of 100 plaques. One-way ANOVA indicated that the virus (***, *P* < 0.001) had a significant impact on phenotypic observations. (C) Plaque phenotyping. CCB cells were infected with the viruses indicated. At 3 days PI, the cell monolayers were stained with DAPI and observed by epifluorescence microscopy. Representative plaques are shown in image panels corresponding to an area of 387.5 µm^2^. (D) Multistep viral growth assay. CCB cells were infected with the strains indicated at an MOI of 0.05 PFU/cell, and virus in the cell supernatant was titrated at the time points PI indicated. Time 0 represents the virus recovered from the cells by PBS washing at the end of the 2 h inoculation period. Data are presented as the mean ± SEM of triplicate measurements. (E) Plaque size assay. CCB cells were infected as described in Section 2, and plaque areas were measured at the indicated time points PI. Data presented are the mean ± SEM for duplicate measurements of twenty randomly selected isolated plaques. Two-way ANOVA indicated that genotype (***, *P* < 0.001) and time PI (***, *P* < 0.001) had significant impacts on growth kinetics (D) and plaque size (E). Multiple comparisons within each time point (Tukey test) indicated that genotypes had a significant impact on growth kinetics (D) and plaque size (E) at most time points. (F) Growth competition in cell culture. CCB cells were coinfected in triplicate with the two viruses indicated (MOIs of 0.01 and 0.09 PFU/cell for FL mCherry rec ORF131-A and FL EGFP rec ORF131-T, respectively). At 48 h PI, the cell supernatants were used to inoculate fresh CCB monolayers. Virus was thus passaged five times. The relative abundance of the two viruses in each sample used as inoculum was quantified by plaque assay as described in Section 2. The results are presented as the mean relative proportion + SEM determined for each virus. Two-way ANOVA indicated that virus (***, *P*< 0.001) and passage number (***, *P*< 0.001) had significant impacts on the proportion of each virus. Linear regression and correlation analyses for the reduction of FL EGFP rec ORF131-T according to passage number are shown in the left part of the panel. The right part of the panel presents an epifluorescence image with the overlay of DAPI, mCherry, and EGFP channels from one of the monolayers infected with the supernatant collected at the fifth passage. Scale bar, 1 mm. Two randomly selected ROIs were magnified, and intensity plots for the two channels were determined along the lines indicated. Scale bar, 200 µm.

#### 2.5.1 Production of the FL rev and FL rev WT ORF27 viruses

As shown in Fig. 2A, the truncated *ORF27* encoded by the FL BAC plasmid was replaced by the WT *ORF27* encoded by CyHV-3 strain M3 to generate the FL BAC ORF27 WT BAC plasmid. The FL BAC and FL BAC ORF27 WT BAC plasmids were then co-transfected into CCB cells together with the pGEMT-TK vector, as described previously (Costes et al. 2008), to generate the FL rev and FL rev WT ORF27 viruses encoding WT *ORF55*.

#### 2.5.2 Production of the FL EGFP rec ORF131-A and FL mCherry rec ORF131-A viruses

As shown in Fig. 2A, the FL EGFP rec ORF131-A virus was produced by transfecting the FL BAC plasmid into CCB cells. The FL mCherry rec ORF131-A virus was produced by co-transfecting the FL BAC plasmid together with a recombination cassette to replace the EGFP coding region by a mCherry coding region. The resulting FL mCherry rec ORF131-A virus was plaque-purified on the basis of red fluorescence, as described previously (Boutier et al. 2015a).

#### 2.5.3 Production of the FL EGFP rec ORF131-T and FL mCherry rec ORF131-T viruses

As shown in Fig. 2A, these viruses were produced by co-transfection of the FL BAC ORF131 Del *galK* plasmid, which is deleted for *ORF131* and thus unable to reconstitute virus (Vancsok et al. 2017), together with recombination cassettes. Thus, the FL EGFP rec ORF131-T virus was generated using the ORF131-183T cassette, which consisted of the ORF131 183T genovariant (amplified from CyHV-3 strain E) flanked by 500 bp upstream and downstream of *ORF131* in CyHV-3 strain FL. This virus was modified further to produce the FL mCherry rec ORF131-T virus by using a mCherry cassette to replace the *EGFP ORF*. The mCherry rec ORF131-T virus was plaque-purified on the basis of red fluorescence, as described previously (Boutier et al. 2015a).

#### 2.5.4 Production of the FL rev ORF131-A and FL rev ORF131-T viruses

As shown in Fig. 6A, these viruses were produced by co-transfection of the FL BAC or FL BAC ORF131 Del *galK* plasmid and the recombination cassettes described above targeting *ORF131* and *ORF55*.

#### 2.5.5 Production of the FL rev luc ORF131-T

A recombinant strain encoding the ORF131 183A genovariant and a firefly luciferase (luc) expression cassette inserted in the intergenic region between *ORF136* and *ORF137* was reported earlier (Costes et al. 2009). This strain is called FL rev luc ORF131-A in Fig. 6A. In the present study, we produced a strain, hereafter called FL rev luc ORF131-T, which differed from the former only by the SNP *C225791T*, so that it encodes the ORF131 183T genovariant (Fig. 6A). This strain was produced by homologous recombination induced in eukaryotic cells by transfection of the pGEMT-136LUC plasmid (Costes et al. 2009) and infection with the FL rev ORF131-T strain. The recombinant strain was plaque-purified using an IVIS Spectrum *in vivo* imaging system (PerkinElmer). The recombinant strain was cloned by three successive steps of plaque picking. Subsequently, the genotype of the recombinant strain was confirmed by genome sequencing.

### 2.6 Genetic characterization of CyHV-3 recombinants

The molecular structures of all recombinant strains were confirmed by monitoring SacI restriction fragment length polymorphism (RFLP) by agarose gel electrophoresis and full-length genome sequencing as described previously (Boutier et al. 2015a).

### 2.7 Multistep growth curves

Triplicate cultures of CCB cells were infected at a multiplicity of infection (MOI) of 0.05 plaque-forming unit (PFU)/cell. Viral inoculums were back-titrated to ensure that the cells were infected with the expected number of PFU. After an incubation period of 2 h, the cells were washed with PBS (the virus recovered in the PBS is reported as time 0) and overlaid with Dulbecco’s modified essential medium (DMEM; Sigma) containing 4.5 g glucose/l and 10 per cent (v/v) FCS. The supernatant was removed from the infected cultures at successive intervals and stored at −80°C. Infectious virions were titrated by duplicate plaque assay in CCB cells as described previously (Ouyang et al. 2013).

### 2.8 Plaque size assay and syncytial plaque assay

CCB cells grown in six-well plates were infected with CyHV-3 strains at 200 PFU/well. After an incubation period of 2 h, the cells were overlaid with DMEM containing 10 per cent FCS (v/v) and 1.2 per cent (w/v) carboxymethylcellulose (CMC; medium viscosity; Sigma), which prevents the spread of the virus through the extracellular medium, facilitating the formation of isolated plaques (Vanderplasschen et al. 1993). At various times postinfection (PI), the cells were fixed in PBS containing 4 per cent PAF at 4°C for 15 min and then at 20°C for 10 min. Images of the monolayers were captured using a Nikon A1R epifluorescence microscope. Plaque size was determined by measuring the area of twenty independent plaques in duplicate wells using ImageJ software (Abramoff, Magalhães, and Ram 2004). Syncytium formation was quantified by examining 100 plaques in triplicate wells.

### 2.9 Live-cell imaging and analysis

**Figure 3.**
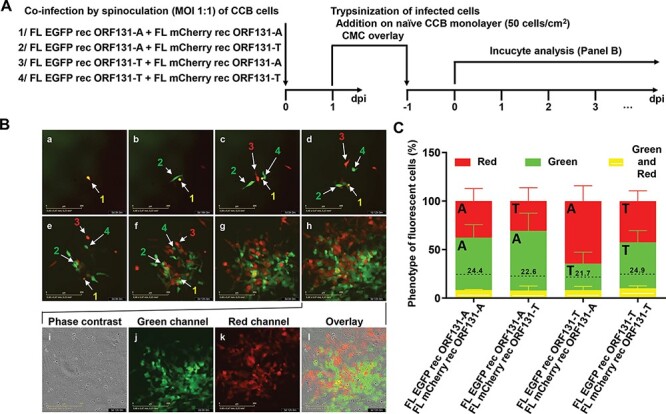
Inhibition of superinfection *in vitro* observed during plaque development. (A) Flow chart of the experiment. CCB cells were coinfected by spinoculation (1,000 × g for 2 h at 25°C) with the pairs of recombinants indicated (MOI of 1 PFU/cell for each recombinant). At 1 day PI, the cells were trypsinized and added to fresh CCB monolayers at a density of 50 cells/cm^2^ and then overlaid with CMC. Doubly infected cells (identified on the basis of the expression of both mCherry and EGFP) and subsequent spread of viral infection were monitored every hour for 7 days by time-lapse microscopy. (B) Images of coinfection with FL EGFP rec ORF131-A and FL mCherry rec ORF131-A, which were typical of all four pairs of viruses. Images a–h illustrate the same area of the monolayer at the time points PI indicated. The numbers represent the order of appearance of cells expressing fluorescence, and the colors of the numbers correspond to the marker responsible (red, mCherry; green, EGFP; and yellow, both). Images i–l illustrate the monolayer in panel h presented in phase contrast, green fluorescence, red fluorescence, and an overlay. A movie covering the entire observation period is provided as supplemental material (Movie S1). (C) Relative proportions of singly and doubly infected cells in plaques derived from isolated cells infected with both viruses, according to the marker responsible (see earlier). A, ORF131 183A genovariant; T, ORF131 183 T genovariant. The dotted lines represent the percentage of doubly infected cells expected in the absence of inhibition of superinfection. These values were calculated by multiplying the observed relative proportions of singly infected cells expressing EGFP or mCherry. The data represent the mean + SEM based on the analysis of three plaques, each containing 300–2,000 cells.

**Figure 4.**
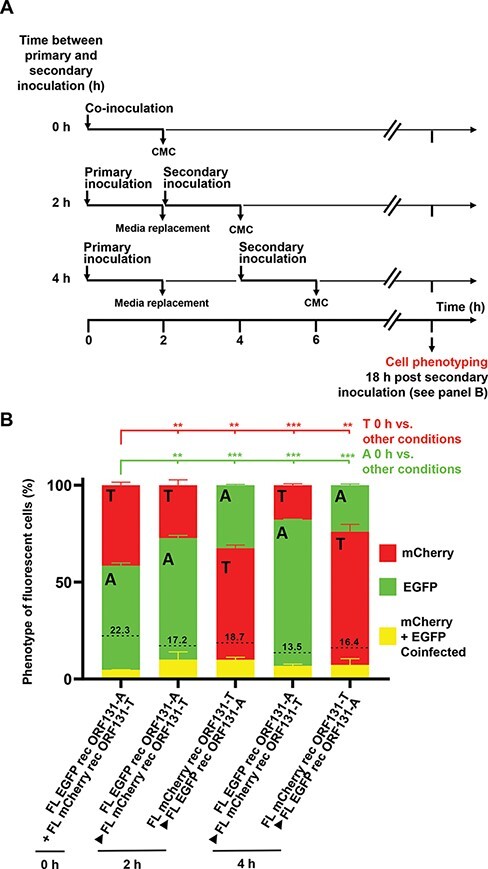
Inhibition of superinfection *in vitro* according to the time post primary infection. (A) Flow chart of the experiment. CCB cells were coinfected (0 h) or infected in sequence (2 and 4 h) by incubation with medium containing (2 h at 25°C) the pairs of recombinants indicated (MOI of 0.25 PFU/cell for each recombinant). Eighteen hours after having been overlaid with CMC, cells were phenotyped by fluorescent microscopy as described in Fig. 3 legend and in Section 2. (B) Relative proportions of singly and doubly infected cells according to the marker responsible. A, ORF131 183A genovariant; T, ORF131 183T genovariant. The dotted lines represent the percentage of doubly infected cells expected in the absence of inhibition of superinfection. These values were calculated by multiplying the observed relative proportions of singly infected cells expressing EGFP or mCherry. The data represent the mean + SEM based on the analysis of duplicate wells (**, *P*< 0.01; ***, *P*< 0.001).

CCB cells cultured in twenty-four-well plates were imaged hourly using an IncuCyte Zoom HD/2CLR time-lapse microscopy system (Sartorius) equipped with an IncuCyte Zoom 20× Plan Fluor objective (Sartorius). Images were collected in phase contrast and in the green (EGFP) and red (mCherry) channels. Stacks of images were exported in tagged image file format using the time plot function. Fluorescence analysis was adapted to set the basic analytical parameters with adaptive segmentation (https://ki.se/en/media/111654/download). The green calibrated unit (GCU) and red calibrated unit (RCU) threshold values were set at 2.0 for the analyses presented in Fig. 3. For the analyses related to Fig. 4, the GCU and RCU threshold values were set at 2.00 and 1.00, respectively. These parameters were set up based on the analysis of specimens infected with EGFP or mCherry recombinants to maximize the detection of each fluorochrome in its wavelength window without signal detection in the wavelength window of the other fluorochrome. This approach was used to ensure that cells detected as double positive represented cells coinfected by EGFP and mCherry recombinants and not artifact of fluorochrome spillover.

### 2.10 Fish

The experiments were performed in common carp (*Cyprinus carpio carpio*). Fish were kept in 60 L tanks at 24°C. Water parameters were checked twice per week. Microbiological, parasitic, and clinical examinations were conducted immediately prior to the experiments to ensure that the fish were healthy. All experiments were preceded by an acclimatization period of at least 2 weeks.

### 2.11 Infection of fish with CyHV-3

Infection was carried out either by immersion of uninfected fish in water containing virus or by cohabitation of uninfected fish with infected fish. Fish were inoculated by immersion by placing them in water containing virus for 2 h under constant aeration, with the volume of water adjusted to the number and size of fish to give a biomass of around 10 per cent. At the end of the incubation period, the fish were returned to 60 L tanks. Virus inoculum was back-titrated to ensure that the fish were infected with the expected number of PFU. For inoculation by cohabitation, infected fish were released into a tank of uninfected fish at a ratio of four infected fish per twenty uninfected fish (Fig. 6C). After a cohabitation period of 2 days, the primary infected fish were removed from the tank. The morbidity rate was determined based on daily observation of each tank. To facilitate the observation of each fish, short videos were recorded and analyzed. Fish expressing at least one of the following clinical signs were considered as exhibiting morbidity: apathy, folding of the dorsal fin, hyperemia of the skin, increased mucus secretion, skin lesions, suffocation, erratic swimming, and loss of equilibrium. Fish reaching the end points defined in the bioethics protocol (protocol no. 2130) were euthanized.

### 2.12 Ethics statement

The experiments, maintenance, and care of fish complied with the guidelines of the European Convention CETS 123. The animal studies were approved by the local ethics committee of the University of Liège, Belgium (laboratory accreditation no. 1610008, protocol no. 2130). All efforts were made to minimize suffering.

### 2.13 Quantification of viral genome copies in fish organs by real-time PCR

Viral genome copies were quantified by real-time TaqMan qPCR, as described previously (Boutier et al. 2015a), by amplifying fragments of CyHV-3 *ORF89* and the carp *glucokinase* gene. The limit of detection was defined as the mean Cq for no template control (NTC) samples minus 1 SD. Reactions were performed using a CFX96 Touch real-time PCR detection system (Bio-Rad) with detection in the FAM channel, and the analysis was performed using CFX Manager 3.0 software (Bio-Rad). The primers and probes used are listed in Table 1.

### 2.14 RNase H-dependent PCR for SNP genotyping

The rhAmp SNP genotyping system (Integrated DNA Technologies) was used with plasmids encoding the ORF131 183A (A at position 225791 of CyHV-3 FL strain genome (GenBank Accession MG925487.1)) or ORF131 183T (T at position 225791 of CyHV-3 FL strain genome (GenBank Accession MG925487.1)) genovariants as references. Primers used for construction of these plasmids and for RNase H-dependent PCR (rhPCR) are listed in Table 1. The standard protocol (www.idtdna.com/rhAmp-SNP-protocol) was used for rhPCR in a 5µl volume, with modifications to improve the genovariant specificity (Fig. S1). A target enrichment step was used prior to rhPCR to aid the relative quantification of genovariants in samples with low viral content (Fig. S1). The limit of detection was defined as the mean Cq for NTC samples minus 1 SD. The rhPCR assays were performed using a CFX96 Touch real-time PCR detection system with the detection in the FAM (for ORF131 183A) and HEX (for ORF131 183 T) channels, and the data were analyzed using CFX Manager 3.0 software. The primers used are listed in Table 1.

### 2.15 *In vivo* bioluminescence imaging

Firefly luciferase was imaged using an IVIS Spectrum *in vivo* imaging system (PerkinElmer), as described previously (Costes et al. 2009; Boutier et al. 2015a). Fish were analyzed *in vivo* lying on their right and left sides. Images were acquired using a field view of C, a maximum autoexposure time of 1 min, a binning factor of 4, and an f/stop of 1. The relative intensities of bioluminescence and scales were determined automatically and represented as a pseudo-color image ranging from violet (least intense) to red (most intense) using Living Image 4.7.3 software. Regions of interest (ROIs) were drawn manually by surrounding the body outline, and the average radiance (p/sec/cm^2^/sr) was taken as the final measure of the bioluminescence emitted over the ROI.

### 2.16 Statistical analysis

**Figure 5.**
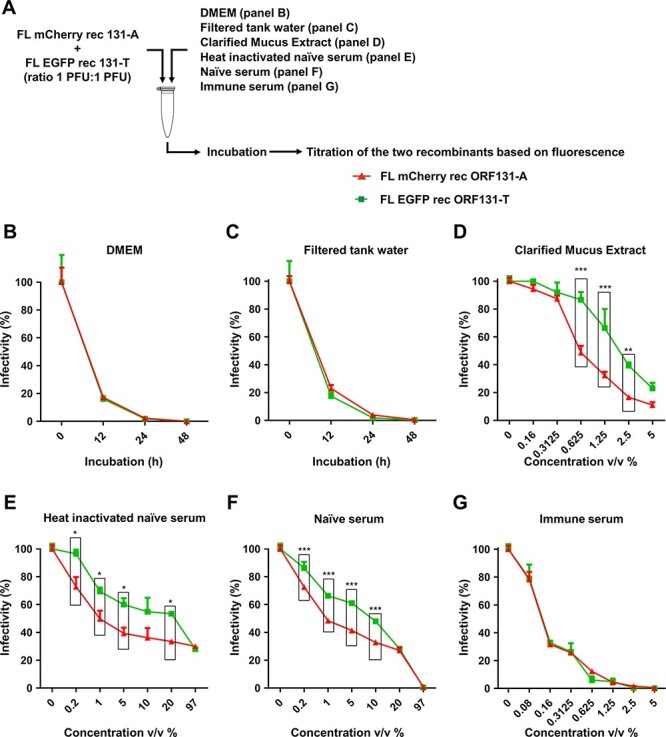
Effects of ORF131 genovariants on virion infectivity. (A) Flow chart of the experiment. Viruses expressing red (ORF131-A) or green (ORF131-T) fluorescence were mixed at equivalent infectivity levels (1PFU:1PFU, with 6 × 10^5^/ml of each genovariant) in a single tube and incubated at 25°C in the indicated medium compositions (panels B–G). Infectivity was measured with respect to negative controls, i.e. 0 h incubation (panels B and C) or 0 per cent v/v of the agent tested (panels D–G, 2 h incubation period). (C) Water taken from a mock-infected fish tank and sterilized by filtration through a 0.22-µm filter. (D) Clarified mucus extract (CME) produced from uninfected fish, as described previously (Raj et al. 2011). The CME used contained 5.0 µg/µl protein. (E) Inactivated naive carp serum prepared by heating naive carp serum at 56°C for 30 min. (F) Naive carp serum. (G) CyHV-3 immune carp serum collected from fish vaccinated using an attenuated recombinant vaccine (Boutier et al. 2015a). Data presented are the mean + SEM for triplicate measurements (representing the counting of 200–300 PFU for the reference). Two-way ANOVA was used for the statistical analysis, taking (B–C) ORF131 genovariant and incubation time or (D–G) ORF131 genovariant and agent concentration as variables (*, P < 0.05; **, P < 0.01; ***,  *P*< 0.001). With the exception of genotypes in panels B, C, and G, all variables (***, *P*< 0.001) were found to have a significant impact on virion stability, as measured by infectivity (%).

**Figure 6.**
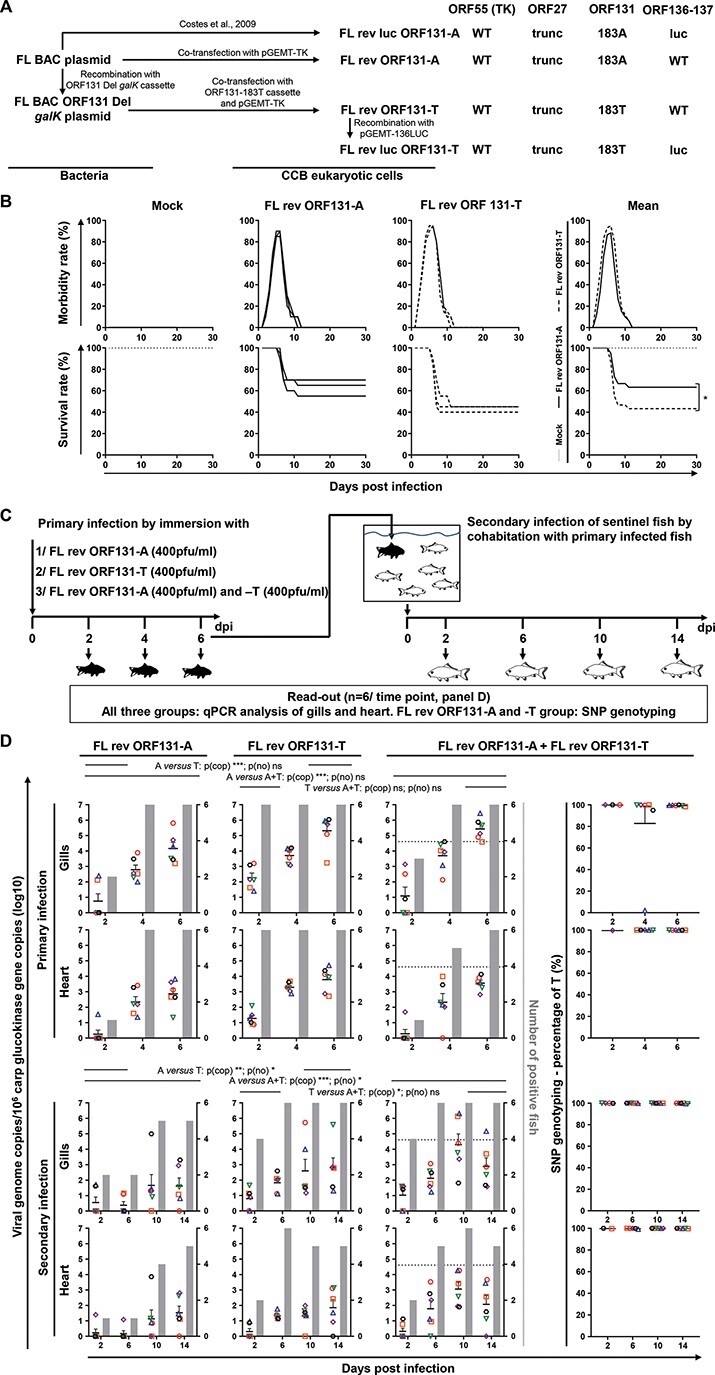
Effects of ORF131 genovariants on virulence. (A) Flow chart illustrating the production of FL rev ORF131-A, FL rev ORF131-T, and FL rev luc ORF131-T strains. The right part summarizes the viral genotypes with respect to *ORF55*, *ORF27*, *ORF131*, and the *ORF136-137* intergenic region. (B) Effect of ORF131 genovariants on virulence. Common carp (triplicate groups with each group containing twenty subjects, mean weight ± SD, 15.1 g ± 3.8 g) were used to compare morbidity and mortality caused by each genovariant. At day 0, the fish were infected for 2 h by immersion in water containing no (mock-infected) or 400 PFU/ml virus. The morbidity rate (top panel) and survival rates (bottom panel) were measured over a period of 30 days PI. Each condition (Mock, FL rev ORF131-A, and FL rev ORF131-T) is presented separately in the first three columns by individual graphs. The last column summarizes the combined data from each condition by mean curves based on three replicates. Survival curves were compared using log-rank tests. (C) Flow chart illustrating the experiment to investigate ORF131 genovariant transmission and competition *in vivo*. This experiment consisted of duplicate tanks for each of the three infection conditions, with each tank containing twenty subjects. The fish were infected by immersion for 2 h in water containing 400 PFU/ml FL rev ORF131-A or FL rev ORF131-T or both (black fish). At 2, 4, and 6 days post primary infection, the primary infected fish were sampled (*n* = 6, with three fish originating from each duplicate tanks) and submitted to qPCR analysis of gills and heart (all three groups). Samples of the coinfected group were also submitted to SNP genotyping. At 6 days post primary infection, some of the remaining infected fish were used (ratio of four infected fish/twenty sentinel subjects) to infect sentinel naive fish (white fish; duplicate groups containing twenty subjects). At the indicated time post secondary infection, sentinel fish (*n* = 6, with three fish originating from each duplicate tanks) were submitted to the same analyses as those applied to primary infected fish. (D) The primary infected fish (top panel) and sentinel fish (secondary infection, bottom panel) were sampled (*n* = 6, with three fish originating from each duplicate tanks) at the indicated times PI and analyzed by qPCR (three columns in the left). Fish sampled from the coinfected groups were also analyzed by ORF131 genovariant genotyping (right column). Throughout this panel, the data obtained for each fish (according to viral strain and time PI) are represented by the same symbol to allow the correlation of the data obtained for the different organs and with the different readouts. For both qPCR and genovariant genotyping assays, the number of viral genome copies is expressed as log_10_ copies per 10^6^ carp glucokinase gene copies. Individual values represent the mean of duplicate measurements for each fish. Mock-infected fish were used as a negative control, and no viral genome copies were detected in these fish. The number of positive subjects among the six fish analyzed is represented by the gray bars. Given the limit of sensitivity of the genovariant assay, the dotted lines in the graphs in the third column represent the minimum detectable viral load (4 × 10^4^ initial copies per reaction) without the need for further target enrichment prior to genovariant genotyping. Samples with viral loads lower than this threshold were submitted to amplification of the *ORF131* region containing the SNP by conventional PCR prior to genovariant genotyping (see Fig. S1 and Table S1). The statistical analysis was performed by combining data from both organs sampled (heart and gills). Combining data from both organs, two-way ANOVA indicated that viruses (***, *P*< 0.001 ) used and time PI (***, *P*< 0.001 ) had significant impacts on viral load. Combining data from both organs and all times PI, post-hoc pairwise Tukey tests indicated that there were significant differences in viral load based on which viruses were used to infect fish (p(cop)). Data on the number of positive fish per group (p(no)) were analyzed in a similar manner using nonparametric tests. Combining data from both organs, a Durbin test indicated that time PI (*, *P*< 0.05 ) had a significant impact on the number of positive fish, but the virus used for each infection only had a significant impact on the number of positive fish during the secondary infection (*, *P* < 0.05). Post-hoc pairwise Wilcoxon tests, combining data from both organs and all times PI, indicated that the numbers of positive fish were significantly lower in the FL rev ORF131-A group compared to other groups but only during the secondary infection (p(no)).

**Figure 7.**
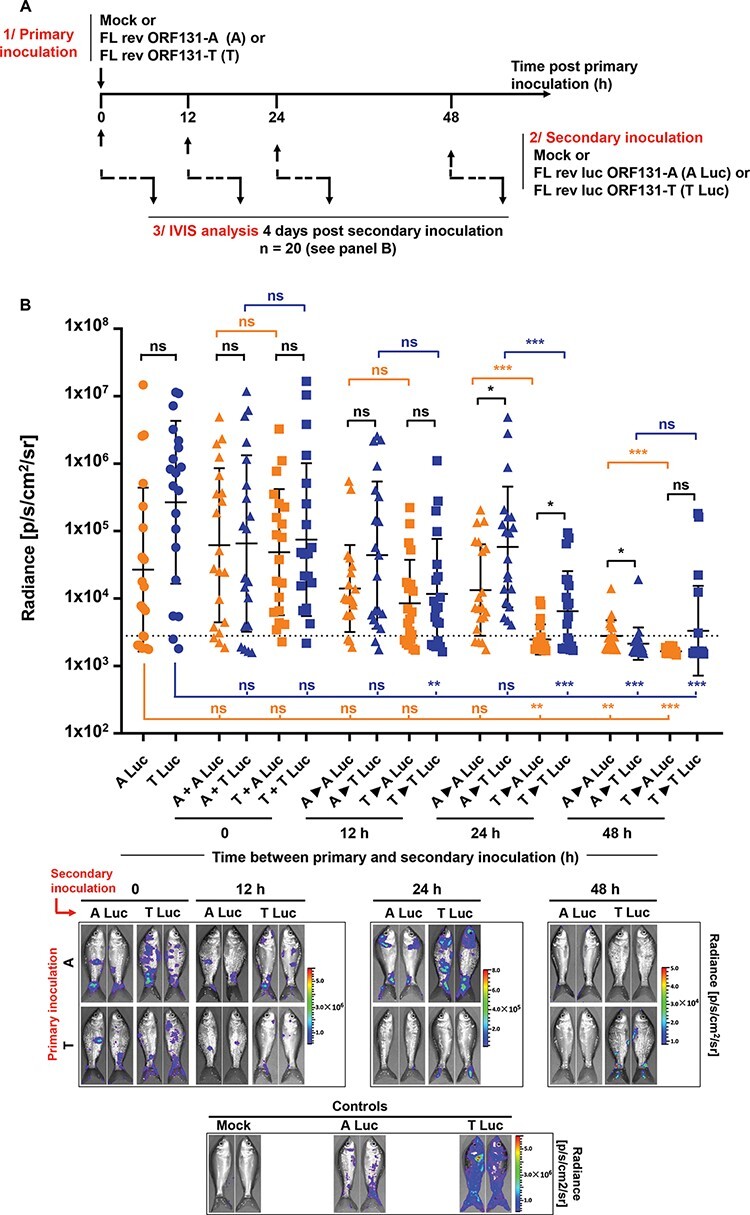
Inhibition of superinfection *in vivo*. (A) Flow chart of the experiment. At the time of inoculation, common carp (mean weight ± SD, 8.71 ± 2.44 g) were mock-infected or infected for 2 h by immersion in water containing 400 PFU/ml FL rev ORF131-A (A) or FL rev ORF131-T (T). At the indicated times post primary inoculation, fish were infected by immersion in water containing 400 PFU/ml FL rev luc ORF131-A (A Luc) or FL rev luc ORF131-T (T Luc), which express luciferase as a reporter. At 4 days post inoculation of luciferase recombinant strains, fish (*n* = 20, consisting of ten fish from duplicate tanks) were imaged for bioluminescent expression on the skin lying on their left or right sides. (B) Average radiance (individual values, mean ± SD) measured on the entire body surface of fish (individual values representing the mean values obtained for the left and right sides of each fish) was used as an indicator of inhibition of superinfection. Triangle and square symbols represent fish primary inoculated with FL rev ORF131-A and FL rev ORF131-T, respectively. Orange and blue colors represent fish secondary infected with FL rev luc ORF131-A and FL rev luc ORF131-T, respectively.The dotted line represents the threshold of positivity, which is the mean + 3 SD of the values obtained for mock-infected fish (data not presented). Bioluminescent data were analyzed by two-way ANOVA (*, *P*< 0.05; **, P < 0.01; ***,  *P*< 0.001). Representative images of IVIS data are presented in the lower part of the figure. Fish with the closest scores to the mean have been selected for image illustration.

Syncytium quantification results (Figs 1B and 2B) were analyzed using one-way ANOVA, and multiple comparisons between groups of interest were made using a post-hoc pairwise Dunnett’s test. Results for viral growth (Fig. 2D), plaque size (Fig. 2E), *in vitro* competition (Fig. 2F), virion infectivity (Fig. 5B–G), viral load as measured by qPCR (Fig. 6D), and bioluminescence measurements (Fig. 7B) were analyzed using two-way ANOVA, and multiple comparisons between groups of interest were made using a post-hoc pairwise Tukey test. Morbidity and survival curves (Fig. 6B) were compared using log-rank tests. These analyses were done using GraphPad Prism 8. The number of fish positive for each ORF131 genovariant (Fig. 6D) was compared by the Durbin test (Durbin 1951), and a comparison between groups of interest was made using a post-hoc pairwise Wilcoxon test (with *P*-values adjusted using the Benjamini–Hochberg method) implemented in R using the R core stats package (R Core Team 2022) and the PMCMR package (Pohlert 2015). The variables used for each omnibus test and multiple comparisons selected for statistical illustrations are described in Section 3 and figure legends, in which the statistical significance for all tests are represented by the following symbols: *, *P* < 0.05; **, P < 0.01; ***, *P* < 0.001; and nonsignificant, ns.

## 3. Results

### 3.1 CyHV-3 strains can be classified into two groups on the basis of syncytial plaque formation

Recently, we performed genomic and biologic comparisons of seven CyHV-3 strains that represent all known CyHV-3 clades (Gao et al. 2018). The comparison of the properties of these strains *in vitro* and *in vivo* revealed a negative correlation between viral growth *in vitro* and virulence *in vivo*. The FL, Cavoy, and T strains were the most fit in cell culture but the least virulent *in vivo*. The opposite was the case for the M3, I, E, and GZ11-SC strains. In the present study, we compared the viral plaque phenotypes of these strains (Fig. 1A and B). In contrast to the strains that were more virulent *in vivo*, the strains adapted to cell culture formed syncytial plaques. Typically, syncytial plaque formation involves fusogenic activity resulting directly or indirectly from cell surface expression of viral glycoproteins (Compton and Schwartz 2017; Kim et al. 2017; Kuny et al. 2020). Therefore, as a means of identifying genetic traits linked to syncytial plaque formation, we compared the sequences of the predicted transmembrane proteins encoded by each strain. This led to the identification of two candidate loci (Fig. 1C). Each syncytial strain encodes a unique, mutated form of *ORF27*, resulting in a truncated protein (Fig. 1D). In addition, each syncytial strain has the same genovariant in ORF131 (183A), whereas the non-syncytial strains have 183T (Fig. 1C and E). Sequence alignments indicated that 183A in the syncytial strains did not have a monophyletic origin and did not emerge by inter-strain recombination. Since we had demonstrated previously that the FL, Cavoy, and T strains were the most fit in cell culture (Gao et al. 2018), we reasoned that syncytial plaque formation may represent a form of adaptation to this environment, as has been reported recently for herpes simplex virus type 1 (HSV-1), a member of the family *Herpesviridae* (Kuny et al. 2020). The results presented earlier demonstrate that CyHV-3 strains can be classified into two groups on the basis of their ability to form syncytial plaques in cell culture. The sequence analysis suggested that the genetic determinants of syncytial plaque formation are located in *ORF27* or *ORF131* or both and that they arose independently in each strain.

### 3.2 The ORF131 183A genovariant determines syncytial plaque formation

Next, we used recombination technologies with the FL BAC plasmid of the CyHV-3 genome to investigate the contribution of the *ORF27* and *ORF131* mutations to the syncytial phenotype. This plasmid and the virus that is subsequently produced from this plasmid (FL rev; Fig. 2A) encode a truncated ORF27 and the ORF131 183A genovariant. Using the same FL BAC plasmid, we produced a second virus (FL rev WT ORF27; Fig. 2A), in which *ORF27* had been reverted to WT. Like FL rev, this virus produced syncytia in cell culture (Fig. 2B), thus demonstrating that the truncation in *ORF27* was not essential for syncytial plaque formation. We then produced four more viruses (FL EGFP rec ORF131-A, FL mCherry rec ORF131-A, FL EGFP rec ORF131-T, and FL mCherry rec ORF131-T; Fig. 2A) to investigate the role of the *ORF131* SNP in syncytial plaque formation. These viruses encoded a truncated ORF27, a fluorescent reporter cassette (EGFP or mCherry, exhibiting green or red fluorescence, respectively; Fig. 2C), and the ORF131 183A or 183T genovariant. The two strains with the 183A genovariant formed syncytia, whereas the two strains with the 183T genovariant did not (Fig. 2B). These results demonstrated that the ORF131 183A genovariant is responsible for syncytial plaque formation.

### 3.3 The ORF131 183A genovariant confers higher fitness *in vitro* than the 183T genovariant

The differences in growth between CyHV-3 strains in cell culture (Fig. 1C of the present study and Fig. 3 of our previous study (Gao et al. 2018)), and the further genetic characterization of these strains in the present study, suggested that the ORF131 183A genovariant confers higher fitness in cell culture relative to the 183T genovariant. To test this hypothesis, the four viruses mentioned earlier were assessed by multistep viral growth assay and plaque size assay (Fig. 2D and E). Independent of the fluorescent reporter gene, viruses encoding the 183A genovariant exhibited more efficient replication, reaching their peak titer earlier (Fig. 2D) and forming larger plaques (Fig. 2E). Moreover, viruses expressing the same ORF131 genovariant but encoding different fluorescent reporter genes exhibited similar viral growth properties and plaque sizes. These results suggest that the ORF131 183A genovariant, which is present in CyHV-3 strains that are the most fit in cell culture, may represent a mutation that is selected *in vitro*, allowing viruses encoding this genovariant to outcompete the parental virus encoding the 183T genovariant (the only genovariant detected in field isolates). To test this hypothesis, FL mCherry rec ORF131-A and FL EGFP rec ORF131-T were used as a mixture to coinfect CCB cells at MOIs of 0.01 and 0.09, respectively (Fig. 2F). Despite the initial bias in favor of the virus encoding the ORF131 183T genovariant, the relative proportion of viruses encoding the ORF131 183A genovariant increased progressively during passaging, representing the vast majority of virus at passage 5. The microscopy images presented in the right part of Fig. 2F illustrated an observation made during plaque assay aiming to determine the relative abundance of 183A (red fluorescent plaques) and 183T (green fluorescent plaques) genovariants in the samples. These images represent monolayers at low dilutions (resulting in a high degree of plaque confluency). While these low dilutions could not be used to count individual plaques to determine the titer of each genovariant, they did reveal the absence of double-positive cells at the borders between plaques formed by the viruses expressing either mCherry or EGFP. This observation stimulated the experiments presented in the next two figures.

### 3.4 CyHV-3 induces interference *in vitro*

If superinfection (one virus establishing a replicative infection in a cell already infected with another) is not inhibited, most coinfected cells should arise by this means, simply because such events should occur more frequently than the simultaneous entry of two different viruses into the same cell. The results in Fig. 2F suggested that the inhibition of superinfection occurs independently of the ORF131 genovariant encoded. This phenomenon of interference (introduced earlier) could result from different mechanisms within the already infected host cells, such as the inhibition of viral entry, the inhibition of capsid transport and unfolding, blockage of genome entry into the nucleus, or reduced access of the superinfecting genome to already saturated replication site into the nucleus (Kobiler et al. 2010). To further support the hypothesis that CyHV-3 infection induces interference against a superinfecting virus, we performed the experiment described in Fig. 3. Cells were first coinfected by spinoculation with a pair of viruses expressing EGFP or mCherry and representing all possible combinations of the two ORF131 genovariants (183A/183A, 183A/183T, 183T/183A, and 183T/183T; Fig. 3A). In order to monitor the progeny originating from individual cells, infected monolayers were trypsinized at 24 h PI and used to seed uninfected cell cultures. The monolayers were then overlaid with CMC to induce the formation of plaques and placed in a time-lapse microscopy system for long-term observation of epifluorescence due to expression of EGFP (green) or mCherry (red). Regardless of the combination of ORF131 genovariants, the spread of virions from cells coinfected with both viruses (expressing both EGFP and mCherry and therefore yellow) did not lead to the formation of plaques consisting of many coinfected cells. On the contrary, it led to plaques consisting predominantly of a mosaic of singly infected cells (expressing either EGFP or mCherry and therefore green or red in appearance). A few coinfected cells were observed, and these were isolated within the mosaic plaques rather than concentrated where the zones expressing either EGFP or mCherry met (Movie S1). Moreover, the frequency of coinfected cells was systematically lower than the expected frequency under simulations of lack of inhibition of superinfection (Fig. 3C). These observations suggested that CyHV-3 infection *in vitro* is rapidly followed by interference reducing successful superinfection. This conclusion suggests that the small number of coinfected cells observed is the result of simultaneous infection, rather than superinfection. The inhibition of superinfection may contribute to the selection of genovariants conferring higher fitness in a population of genetically heterogeneous viruses. We also observed that, again regardless of the viruses involved, infected cells migrated within the monolayer during the early stage of infection (see the relative position of numbered cells in Fig. 3B in relation to time PI, Movie S1).

To test further the ability of CyHV-3 infection to induce interference against superinfection of already infected cells, we performed the experiment described in Fig. 4. This experiment involved simultaneous or delayed infection of a monolayer (i.e. infection with one genovariant and then infection with another after) by the FL EGFP rec ORF131-A and FL EGFP rec ORF131-T strains. The result of this experiment further supported the observation that a primary infection of a monolayer reduces its ability to be superinfected by a second virus and that this phenomenon increases with the length of delay between the first and second infections. Importantly, these data also highlight that this phenomenon applies to both ORF131 genovariants.

### 3.5 Effects of ORF131 genovariants on virion infectivity

The observation that strains of CyHV-3 adapted to cell culture environments encode the ORF131 183A genovariant, whereas field strains encode the 183T genovariant, suggested that the former confers a higher fitness in cell culture and the latter confers a higher fitness *in vivo*. To further test the latter hypothesis and to understand the basis of the fitness, we investigated the effects of the genovariant on virion infectivity over time and on sensitivity of virions to neutralization by epidermal mucus and serum immune factors, using FL mCherry rec ORF131-A and FL EGFP rec ORF131-T. In order to avoid potential artifacts resulting from variations between virus preparations (e.g. relative abundance of cell debris or non-infectious particles), we used the approach depicted in Fig. 5. This had the benefit of facilitating the comparison of viruses encoding the two genovariants after mixing them (Fig. 5A). Tests of virion infectivity over time in culture medium (DMEM) and filtered tank water revealed no significant difference between the viruses, and most of the infectivity was lost from both viruses by 12 h of incubation (Fig. 5B and C). In contrast, neutralization assays performed with clarified mucus extract, heat-inactivated naive serum, or naive serum revealed a higher infectivity for virions encoding the 183T genovariant (Fig. 5D–F). However, a difference was not observed in neutralization assays performed using immune serum (Fig. 5G). These results suggested that the 183A and 183T genovariants express comparable stability of infectivity in culture medium and water. Importantly, however, they also suggested that the 183T genovariant is more resistant than the 183A genovariant to neutralizing innate immune factors present in epidermal mucus and serum.

### 3.6 The ORF131 183T genovariant confers higher fitness *in vivo* than the 183A genovariant

Having investigated the relative fitness of each ORF131 genovariant *in vitro*, we subsequently also compared the relative fitness *in vivo*. All CyHV-3 recombinants used up to this point in the study encoded a truncated version of the TK gene. While this was previously shown to have no effect on the growth of CyHV-3 *in vitro*, it does reduce virulence slightly *in vivo* (Costes et al. 2008). Consequently, using the strategy described in Fig. 6A, we produced two viruses encoding WT *ORF55* (encoding TK), each with a different version of the ORF131 genovariant (183-A or -T), referred to as FL rev ORF131-T and FL rev ORF131-A, respectively. The structures of the recombinants were confirmed by combined SacI RFLP and full-length genome sequencing (data not shown). The virulence of these two viruses was compared *in vivo* through inoculation of fish by immersion in water containing virus, thereby mimicking natural infection (Fig. 6B). In both infected groups, fish developed CyHV-3 disease, with the intensity and kinetics of appearance of clinical signs being similar for the two viruses. However, FL rev ORF131-T induced significantly higher mortality than FL rev ORF131-A (Fig. 6B). This suggested that the SNP in *ORF131* that differentiates the two viruses also affects virulence *in vivo*. To test this hypothesis, we compared the virulence of the two viruses further by comparing viral loads in the fish by qPCR. Furthermore, the transmission of the viruses from infected fish to uninfected cohabitant sentinels was also investigated (Fig. 6C and D). In addition to comparing the viruses in separate infections conducted in parallel, we also investigated coinfections where fish were inoculated with equal amounts of both viruses, thus allowing the relative fitness of each virus *in vivo* to be established. This necessitated the development of a method capable of specifically, sensitively, and accurately quantifying the relative abundance of the viruses (which differ by a single nucleotide) in biological samples. To reach this goal, we developed an rhPCR assay that was capable of measuring the relative proportions of the viruses in a dynamic range of 10–90 per cent (Fig. S1).

Fish infected by immersion with FL rev ORF131-T exhibited higher viral loads than fish infected with FL rev ORF131-A. Fish coinfected with both viruses expressed higher viral loads than fish infected with FL rev ORF131-A alone, and the loads were similar to those in fish infected with ORF131 183T alone (Fig. 6D, upper panel). The rhPCR analysis of coinfected fish for both organs tested (gills and heart) revealed lower signals relative to qPCR, due to steps taken to increase the specificity (Fig. S1). However, for all but one fish, the vast majority, if not all, of the detectable signal consisted of FL rev ORF131-T target (Fig. 6D, right column, two upper panels). No significant differences were observed between the primary infected groups in terms of the number of positive fish (Fig. 6D). These results indicate a higher fitness of the ORF131 183T genovariant *in vivo*.

We also compared the ability of the two viruses to spread from infected fish to cohabitant naïve fish. The groups of fish initially infected with FL rev ORF131-T or coinfected with a mixture of this virus and FL rev ORF131-A induced a higher number of positive fish than those infected with FL rev ORF131-A alone (Fig. 6D, lower panels). The analysis of infected cohabitant fish by rhPCR did not result in the detection of the 183A genovariant, suggesting its lower abundance relative to 183T or its absence in the sample. These results suggest that, although FL rev ORF131-A is able to replicate and transmit in *in vivo*, it is less fit than FL rev ORF131-T in this environment, as indicated by separate infections. ORF131-T, having greater fitness *in vivo* (i.e. less susceptibility to innate defenses), also represents more of the viral load during coinfections and thus should eventually outcompete the ORF131-A genovariant in this environment.

### 3.7 Fish infected by CyHV-3 rapidly become resistant to superinfection, and viruses encoding the ORF131 183T genovariant induce resistance more rapidly than viruses encoding the ORF131 183A genovariant

The results described earlier suggested that FL rev ORF131-T may reduce the fitness of FL rev ORF131-A *in vivo*. One possible hypothesis is that the faster and more efficient infection of FL rev ORF131-T in infected fish, combined with more efficient spread to uninfected fish, induces an innate immune response and impairs the entry and replication of FL rev ORF131-A. However, the results in Fig. 6 are based on viral genome copies only, and the approach was not suitable for comparing viral replication in the skin, which plays key roles in CyHV-3 pathogenesis by acting as the major portal of entry, the initial site of viral replication, and then as a secondary site of replication associated with viral egress from infected fish (Costes et al. 2009; Boutier et al. 2015a). Thus, the nature of this *in vivo* competition was scrutinized further using luc-expressing versions of the genovariants used in Fig. 6, which necessitated the generation of a new FL rev ORF131-T recombinant (Fig. 7A).

Fish were first mock-infected or infected with one of the two genovariant viruses, and the mock-infected fish forming the control group (Fig. 7A). The fish were then superinfected at various time points after primary infection with either FL rev luc ORF131-A or FL rev luc ORF131-T (Fig. 7A) expressing firefly luciferase as a reporter. Infection by these viruses was monitored by bioluminescent imaging 4 days after superinfection (Fig. 7A). Regardless of the ORF131 genovariant used for the primary inoculation or the secondary inoculation, infected fish expressed reduced sensitivity to superinfection at 48 h after the primary inoculation. Interestingly, at 24 h post primary inoculation with the ORF131 183T genovariant, both the FL rev luc ORF131-A and the FL rev luc ORF131-T exhibited significantly reduced replication (Fig. 7B, 24 h, T►A Luc, T►T Luc), while a significant inhibition was not observed after primary infection with the ORF131 183A genovariant (Fig. 7B, 24 h, A►A Luc, A►T Luc). These data support the hypothesis that replication of the ORF131 183T genovariant induces a faster and/or higher antiviral innate immune response than the infection by the ORF131 183A genovariant. This was further supported by the observation that the bioluminescent signal generated by a defined genovariant was in three out of four comparisons significantly lower after primary infection with the ORF131 183T genovariant than the 183A genovariant (Fig. 7B; 24 and 48 h). Furthermore, in direct comparison between FL rev luc ORF131-A and FL rev luc ORF131-T after no primary infection or the same primary infections, FL rev luc ORF131-T exhibited a higher mean signal in eight out of nine comparisons (two of which show significant differences). Altogether, the results presented earlier support the higher fitness of the ORF131 183T genovariant *in vivo* compared to the ORF131 183A genovariant.

## 4. Discussion

In the present study, we identified syncytial plaque formation *in vitro* as a common trait of CyHV-3 strains adapted to cell culture. The genetic determinism of this trait was linked to an SNP in *ORF131*, which encodes an essential type 1 virion membrane glycoprotein. Viruses differing only by this SNP (*C225791T* mutation that results in the ORF131 183A and 183T genovariants) were compared for their fitness *in vitro* and *in vivo*. The ORF131 183A genovariant associated with syncytial plaque formation was more fit *in vitro* but the less fit *in vivo* and *vice versa*. The identification of such variants, distinguishable by genotype and phenotype and lying on the opposite ends of the fitness spectrum in each environment, created a unique and valuable opportunity to study the competition between variants in these different environments under controlled conditions. Importantly, for the first time, this allowed us to gain an insight into the process of directional selection between CyHV-3 variants. Experiments involving coinfection by both viruses *in vitro* and *in vivo* showed that the more fit virus contributed to the purifying selection of the less fit virus by outcompeting the latter and that an intrinsic ability to stimulate interference preventing efficient superinfection (at a cellular and/or host level) may act as a means to accelerate this process.

The results of this study stimulated semantic thinking about the concept of genovariants. Does it apply to all genovariants that appear during the viral replication even if they do not persist in the system? Or, is it restricted to genetic mutants that appear in a system and are later able to persist in this system? Data generated in the present study are compatible with both interpretations. Indeed, there is no reason why the mutation leading to the SNP *225791TC* would be restricted to only occurring *in vitro*. This implies that the 183T genovariant (the only detected genovariant in field isolates) should also generate 183A mutants when replicating *in vivo*. Notably, despite the fact that 183A mutants have never been identified in field isolates, our study still demonstrated (using a laboratory model) that the 183A genovariant can replicate and transmit between fish although it exhibits less fitness *in vivo* relative to the 183T genovariant. However, as 183A mutants would always emerge in populations vastly dominated by the 183T genovariant, they should be immediately subjected to negative selection during outbreaks, due to their inferior fitness. Furthermore, according to our data, in a scenario where the 183A genovariant could persist as the only variant, the appearance of the 183T genovariant through mutation should also lead to the negative selection of the former, thus explaining its absence among field isolates, despite its ability to propagate *in vivo*. The opposite phenomenon must have occurred *in vitro*, thus explaining why we observed the 183A genovariant in isolates that have undergone extensive *in vitro* passaging (see isolate information in Gao et al. (2018)). Given that there is no monophyletic relationship between the highly passaged isolates that exhibit the ORF131 183A genovariant (compare Fig. 1 of Gao et al. (2018) and Fig. 1 of the present manuscript), it implies that these ORF131 183A genovariants originated from independent mutation of the 183T genovariant (the initial field isolate) and then outcompeted the parental 183T genovariant in subsequent passages. Indeed, when growing the ORF131 183T genovariant (representative of field isolates) *in vitro*, even though it was present at nearly ten times the level of the 183A genovariant, the latter rapidly outcompeted the former (Fig. 2F). There is nothing to prevent the ORF131 183T genovariant from emerging again in these cultures via mutation; however, it would not persist to the extent where it would be observed. In conclusion, the association of the ORF131 183A genovariant with cell-adapted populations and the correlation of the ORF131 183T genovariant with field isolates does not imply that these two systems do not generate the opposite genovariant from time to time but rather that the latter cannot persist in the presence of the fitter variant.

Syncytial plaque formation induced by the ORF131 183A genovariant was associated with higher fitness in cell culture (Fig. 2D and E). The association between these two phenotypic traits has been demonstrated previously for members of the family *Herpesviridae*, such as HSV-1, for which point mutations in virion fusion glycoprotein B (gB) induce both traits (Parsons et al. 2015; Kuny et al. 2020). There is no detectable ortholog of gB in alloherpesviruses, and the CyHV-3 ORF131 protein shows no sequence similarity to gB or other herpesvirus proteins with known function. In the case of HSV-1, it has been proposed that the enhanced replication of syncytial viruses in cell culture is a consequence of syncytium formation promoting the spread of infection in the cell monolayer. In the case of CyHV-3, we observed that syncytium formation occurred at the late stages of infection and involved infected cells only. This statement relied on confocal observation of syncytia formed by cells infected with CyHV-3 recombinant strains expressing EGFP as a reporter and stained with DAPI. At the late stages of the CyHV-3 replication cycle, cellular chromatin is marginalized at the periphery of the nucleus, so that infected cells can be easily identified by DAPI staining. For a good example, see Fig. 4 of Diallo et al. (2022). Confocal microscopy analysis revealed that CyHV-3 syncytia contained only nuclei expressing marginalized chromatin and so resulting from the fusion of infected cells at a late stage of infection (data not shown). Consequently, we hypothesized that the higher titer of syncytial CyHV-3 strains likely reflected more efficient virion production or higher infectivity of virions in cell culture.

This study stimulates further research on the roles of ORF131 in the CyHV-3 replication cycle. The association of the ORF131 183A genovariant with the expression of syncytia in cell culture suggests that this protein could be involved in the fusion process and that this genovariant is more fusogenic than the ORF131 183T genovariant. However, alternative hypotheses are also compatible with these observations. For example, ORF131 could be a protein involved in the negative regulation of a CyHV-3 fusion property. According to this hypothesis, the ORF131 183A variant would be a less efficient inhibitor than the ORF131 183T variant. Further studies are required to unravel the function of the ORF131 protein in the CyHV-3 replication cycle *in vitro* and *in vivo*. Of note, it will be interesting to determine whether the phenotype observed for the ORF131 183T genovariant relies on the phosphorylation of the 183T amino acid residue expressed in the extracellular region of the protein. The relevance of this question is supported by studies demonstrating the role of phosphorylation in herpesvirus fusion processes (Oliver et al. 2013; Carmichael et al. 2018).

The observed purifying selection of the ORF131 183A genovariant *in vivo* demonstrated that this genovariant confers less fitness than the ORF131 183T genovariant. The latter also exhibited a higher resistance against the innate immune components of epidermal mucus and serum (Fig. 5D–F), which may also contribute to greater fitness observed *in vivo*. Collectively, these observations provide the scope for further investigation into the role of *ORF131* and the impact of the SNP on its functions. Interestingly, studies on distant alphaherpesviruses also demonstrated that genovariants associated with syncytium formation in cell culture correlated with attenuation *in vivo* (Goodman and Engel 1991; Engel, Boyer, and Goodman 1993; Oliver et al. 2013; Yang, Arvin, and Oliver 2014). The infectious model described in this study provides a unique opportunity to address key questions related to this observation, such as, do strains that form syncytia in cell culture also induce syncytia *in vivo*? and if so, how could syncytia formation *in vivo* impair the viral biological cycle?

Extensive passage of viruses in cell culture has been used as a simple approach for producing live-attenuated vaccine candidates. It allows the amplification and selection of variants encoding randomly acquired mutations in genes essential for virulence *in vivo* but dispensable for replication *in vitro*. The CyHV-3 *ORF131* SNPs exposed a more complex situation in which the 183A genovariant conferred the adaptation to cell culture but reduced virulence *in vivo* (Figs 2 and 6). Recently, we described the rational development of an attenuated recombinant CyHV-3 vaccine using the FL strain as parent (Boutier et al. 2015a). The ORF131 183A genovariant encoded by this strain could explain, at least in part, the limited spread of this virus *in vivo* (Boutier et al. 2015a). This observation also has implications for the future rational design of vaccines against CyHV-1 and CyHV-2, both of which encode *ORF131* orthologs. SNPs within these orthologs may produce phenotypes similar to those described in the present study for CyHV-3.

Our recent evidence supporting a general low degree of diversity among *Cyprinivirus* species clades compared to species clades within the family *Herpesviridae* suggests that a high degree of purifying selection has occurred after species clade divergence (Donohoe et al. 2021). We speculated that this difference could be linked, at least in part, to fundamental differences in biology between members of the two distantly related virus families. The present study on CyHV-3 provides further insights into this previous observation. First, the low occurrence of coinfected cells would inevitably reduce genetic shift (inter-strain recombination). Second, the reduction in the generation of diversity may of course be compounded by the inhibition of superinfection by variants with higher fitness, thus accelerating the purifying selection of less fit variants within populations. Indeed, coinfection experiments using two viruses encoding different ORF131 genovariants showed both *in vitro* and *in vivo* that the more fit virus rapidly contributes to the purifying selection of the less fit virus during lytic infections and that the induction of interference inhibiting efficient superinfection may act as a means of accelerating this process. This interference phenomenon has been described for many years in various organisms from plant to animals (Henle 1950). This concept is frequently used as a collective term to illustrate the diversity of the mechanisms at its basis. It will be very interesting to unravel the mechanisms explaining the interference phenomena observed in the present study both *in vitro* and *in vivo*.

Both ORF131 genovariants induced the rapid inhibition of superinfection *in vitro* (Figs 2F and 3, Movie S1, and Fig. 4). Indeed, the inhibition of superinfection was sufficiently strong and rapid to necessitate the use of spinoculation to establish frequent coinfections (Fig. 3). The inhibition of superinfection *in vitro* at a cellular level has been reported for many viral families (Berngruber, Weissing, and Gandon 2010; Webster, Ott, and Greene 2013; Beperet et al. 2014; Biryukov and Meyers 2018), including members of the family *Herpesviridae* (Chase et al. 1990; Criddle et al. 2016; Provost et al. 2017; Koyuncu, Enquist, and Engel 2021), and can occur as early as 2–6 h PI (Banfield et al. 2003; Meurens et al. 2004). To the best of our knowledge, such rapid and extreme inhibition of superinfection that we observed with CyHV-3 has not been described for other viruses and thus opens up multiple avenues for further investigation into the underlying mechanisms. In contrast to the situation *in vitro*, the inhibition of superinfection *in vivo* took much longer to develop and revealed differences between the ORF131 genovariants, in that the 183T genovariant induced inhibition more rapidly and efficiently. On this note, the preliminary data from our laboratory suggest that the interference observed *in vivo* is a consequence of an antiviral innate immune response expressed in the skin.

Given that the viruses we used to investigate this phenomenon *in vivo* differed by an SNP, the application of rhPCR proved to be a crucial tool for monitoring the progress and nature of purifying selection *in vivo* (Fig. 6D). This method is an important innovation in SNP genotyping (Dobosy et al. 2011). Although rhPCR has been applied recently to qualitative viral SNP genotyping (Nakauchi et al. 2020), to the best of our knowledge, until now, it has not been applied to measuring the proportions of SNP-based genovariants in viral populations. Despite the greater specificity of rhPCR than conventional PCR, we still observed non-specific amplification in the presence of non-target genovariants (Fig. S1A). This may be due to mismatches that reduce, rather than eliminate, *Pyrococcus abyssi* RNase H2 activity (Dobosy et al. 2011) and the fact that Taq polymerase can, albeit inefficiently, extend the mismatched 3ʹ ends of primers (Huang, Arnheim, and Goodman 1992). Given that the relative proportions of genovariants within viral populations can differ over a much greater range than alleles within a single organism (in which the range is determined by zygosity and ploidy), the non-specific amplification may hinder the robust relative quantification. Modifications to reduce non-specificity (Fig. S1B) resulted in a useful dynamic range (Fig. S1C) but also in reduced sensitivity. However, the development of a simple and rational target enrichment process, which preserved the relative proportions of the genovariants present (Fig. S1D), extended the usefulness of the rhPCR assay to samples with low viral loads (provided that both targets are above the limit of detection). The approach developed in the present study can, in principle, be applied to any virus population and therefore may be of wider interest beyond this current study.

In conclusion, the present study indicates that CyHV-3 may have an intrinsic ability to contribute actively to the purifying selection of less fit variants by stimulation of superinfection inhibition at both the cellular and the host levels. Many herpesviruses (or variants thereof) and other viruses also have the ability to inhibit superinfection. However, more widely, our observations demonstrate how the fundamental biology of some (perhaps many) viruses and their hosts may have a profound impact on the degree of diversity that arises within viral populations.

## Supplementary Material

vead003_Supp

## Data Availability

All the data related to this study are presented in the manuscript or in its supplemental materials.
