# Supplementary figures and images for: Virus-induced interference as a means for accelerating fitness-based selection of cyprinid herpesvirus 3 single-nucleotide variants in vitro and in vivo
Source: Virus Evol. 2023 Jan 17;9(1):vead003. doi: 10.1093/ve/vead003 (PMC9936792; doi:10.1093/ve/vead003)

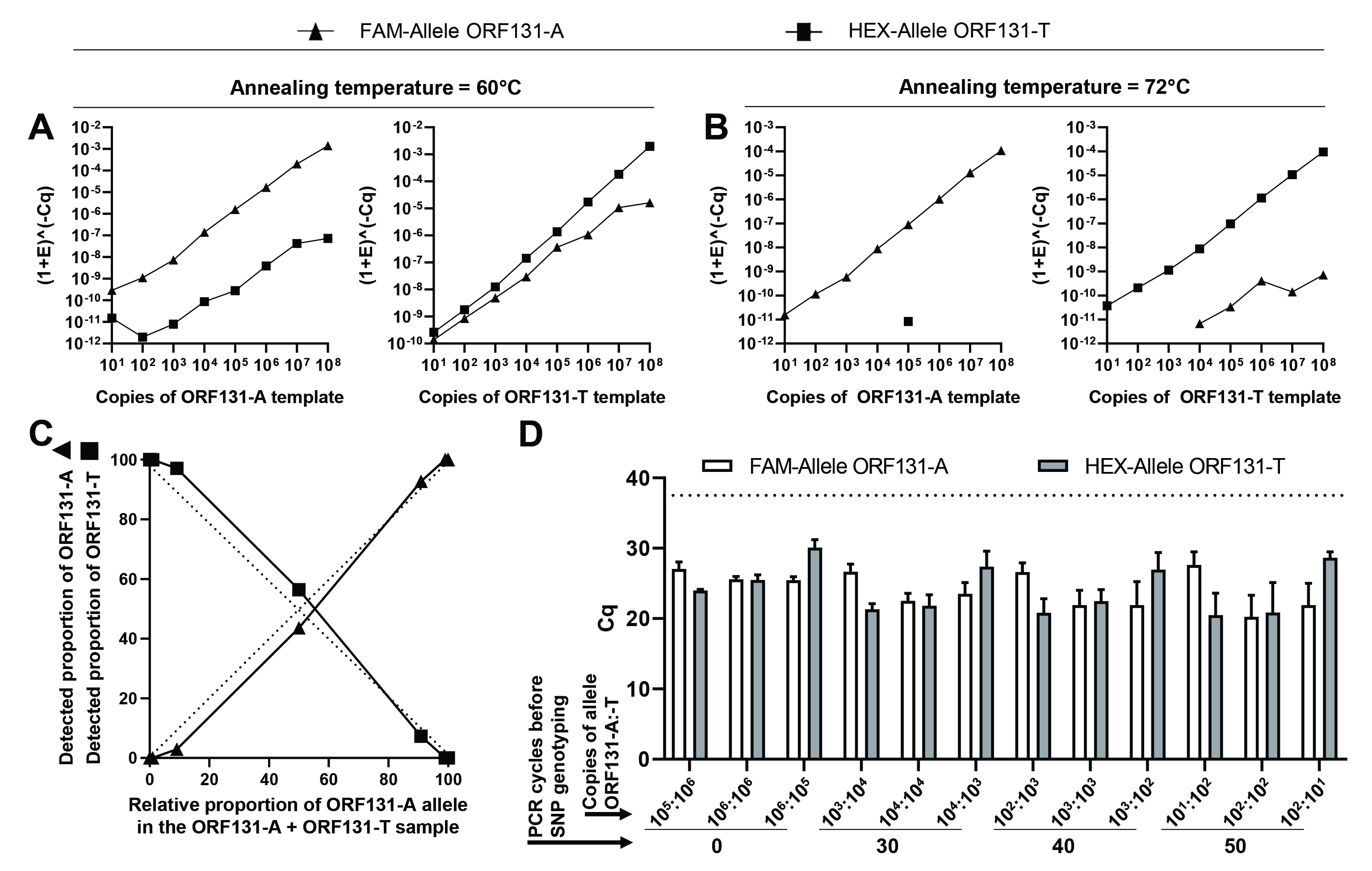

Supplement: vead003_Supp [file vead003_supp.zip › suppl_data/Figure S1 revised Final version.tif]
